# Validation of a Food Frequency Questionnaire for Hispanics

**Published:** 2006-06-15

**Authors:** Gladys Block, Patricia Wakimoto, Christopher Jensen, Shelly Mandel, Robin R Green

**Affiliations:** University of California, Berkeley, School of Public Health, Division of Community Health and Human Development; University of California, Berkeley, School of Public Health, Division of Community Health and Human Development, Berkeley, Calif; University of California, Berkeley, School of Public Health, Division of Community Health and Human Development, Berkeley, Calif; University of California, Berkeley, School of Public Health, Division of Community Health and Human Development, Berkeley, Calif; The David & Alice Jurist Institute for Research, Hackensack University Medical Center, Hackensack, NJ

## Abstract

**Introduction:**

The Hispanic population will grow to comprise one fourth of the U.S. population by 2050. Compared with non-Hispanic whites, Hispanics have disproportionately higher rates of obesity, diabetes, and other diet-related conditions. Valid methods for studying the dietary intake of this group are needed.

**Methods:**

From June through September 2000, we conducted a study of low-income Hispanic men and women (n = 89) who were recruited for a validation study of the Spanish-language food frequency questionnaire used in the Study of Women's Health Across the Nation. The mean age of the participants was 36.8 years, 42% were male, and 92% had been born in Mexico. Three 24-hour dietary recalls provided the reference data. The food frequency questionnaire was administered by interview, with a portion-size graphic to aid in quantitation. The questionnaire asked about diet in the previous 12 months. Mean nutrient values, correlation coefficients, and the sensitivity and specificity for identifying people with intakes of less than the recommended levels were calculated.

**Results:**

Mean energy and macronutrient intake estimates were significantly higher by the food frequency questionnaire than by the 24-hour dietary recalls. Cholesterol, saturated fat, dietary fiber, iron, vitamin A, and percentage of energy from fat were not significantly different by the two methods. The median of unadjusted correlations was 0.52 and of deattenuated correlations was 0.61. The median sensitivity was 0.62, and the median specificity was 0.76.

**Conclusion:**

The Study of Women's Health Across the Nation Spanish food frequency questionnaire appears to be reasonably valid in assessing the dietary intakes of Hispanics. Correlations tended to be higher than those found in other validation studies in Hispanic populations. Interviewer administration of questionnaires may be necessary in this population.

## Introduction

As of 2002, the Hispanic population in the United States was 37.4 million (1), or approximately 13% of the U.S. population. By 2050, the population is expected to grow to 102 million, or 24.5% of the U.S. population (2). Hispanics have disproportionately more diet-related health problems, including obesity (32% higher among Hispanic women than non-Hispanic white women) and diabetes (with 41% greater age-adjusted years of potential life lost than among the non-Hispanic white population) (2). To address the diet-related health disparities, measurement methods for dietary assessment that are valid and appropriate for this population are needed.

Numerous food frequency questionnaires (FFQs) have been developed and tested in the Hispanic population (3-10). However, overall correlations with reference data have been modest. We assessed the validity of one such assessment tool, the Spanish version of the Study of Women's Health Across the Nation (SWAN) questionnaire, in a population of California Mexicans and Mexican Americans. The University of California Committee for the Protection of Human Subjects approved the research, and all participants provided informed consent.

## Methods

### Study population

The study was conducted from June through September 2000. Participants were recruited from community-based organizations primarily serving the low-income Hispanic population in the San Francisco Bay area, including a community center, an organization providing referral services, an organization providing adult education classes (including English as a Second Language classes), and local health clinics — one urban and one semirural. Participants were primarily Mexican and Mexican American and were from both rural and urban areas.

### Study design

Three 24-hour dietary recalls were administered over 2 months. Nutrient estimates based on the mean of the three recalls constituted the reference data against which the test instrument was compared. The test food frequency questionnaire (FFQ) was administered by bilingual interviewers 2 to 3 weeks after the first 24-hour recall. The FFQ measured usual dietary intakes during the previous 12 months.

### FFQ

The SWAN study is a multisite, multiethnic longitudinal study of midlife women in the United States (11). The Newark, NJ, SWAN site enrolled Spanish-speaking participants. The methods used in the development of the SWAN questionnaire have been described previously (12,13). The SWAN FFQs are modifications of the 1995 version of the Block FFQ. In the SWAN study, four versions of the FFQs were prepared (English only and three bilingual versions that combined English with Spanish, Chinese, or Japanese). The core food list, used for all ethnic groups, contained 103 food items identified based on the responses of African American and white participants in the Second National Health and Nutrition Examination Survey (NHANES II) (14). The Spanish-language version included the 103-item core food list, plus nine additional foods appropriate for several Hispanic subgroups. Four of the 103 foods are more important in the diets of Cuban and Puerto Rican Americans than in the diets of Mexican Americans (evaporated and condensed milk; pudding and flan; viandas, plantain, and cassava; and sauces such as mole and sofrito). Five are more important in the diets of Mexican Americans than in the diets of the other two groups (corn tortillas, flour tortillas, cooked green peppers and chile rellenos, avocado and guacamole, and chile peppers and hot chile sauce) (J. Norris, unpublished data, 1996). The additional foods were identified as important in the diets of Hispanics on the basis of their nutrient contribution in NHANES II and the Hispanic HANES (15). In addition, focus groups were conducted among Hispanic volunteers to ensure that the descriptions of the foods captured the forms in which they were commonly eaten.

For most foods, the frequency-of-consumption categories permitted a maximum frequency of "every day." Breads and some snacks could be reported as eaten up to twice per day, and beverages up to five or more glasses or cans per day. Participants were asked about portion size (with four response categories) for each food. For unitary items (e.g., slices of bread), respondents were asked how many were eaten each time. For nonunitary items, the original SWAN study used three-dimensional portion size models; for our study, the Block portion-size graphic was used to facilitate quantification of amounts (16).

### 24-hour dietary recalls

Three 24-hour dietary recalls were administered by experienced bilingual interviewers over 2 months using the multiple-pass method (17). The Food Processor version 7.0 (ESHA Research Inc, Salem, Ore) was used to analyze the nutrients in the foods from the recalls. This version was obtained in 1997; at the time, no information was available on missing values. As of 2000, the first year in which missing value data were available for Food Processor, some nutrients had many missing values. For example, vitamin E was available for only 47% of the foods. Consequently, we only report nutrient values for which the Food Processor program had nutrient content data for at least 90% of the foods on the 24-hour recall database.

### Statistical analysis

Parametric and nonparametric descriptive statistics were calculated. Nutrient variables were log- or square-root transformed if needed to improve normality and reduce skewness. Three participants had a dietary energy intake estimate that was more than three standard deviations above the mean for the respective instrument (one from the recalls and two from the FFQ) and were excluded from the analytic sample. The relationship between the FFQ and the 24-hour dietary recall nutrient values was estimated using Pearson correlation coefficients. For some analyses, correlations were energy adjusted using the residual method (18,19) and the nutrient density method (nutrient/calories) (18). Because results of the two energy-adjustment methods were similar, we report here the more commonly understood density method. For nutrient estimates that include the contribution of vitamin supplements, the correlations are not energy adjusted. We estimated deattenuated correlations to correct for the variability in the recalls (20). We also calculated the sensitivity of the FFQ to identify respondents meeting or failing to meet recommended intakes based on *Dietary Guidelines* *for Americans* (21) or *Dietary Reference Intakes* (22).

## Results

The mean age of the participants was 36.8 years (Table 1), and almost 42% of the analytic sample was male. Participants were born in Mexico (92.1%), South America (2.3%), or the United States (5.6%). Five participants completed fewer than three 24-hour recalls. However, results and conclusions were similar regardless of their inclusion or exclusion, and therefore they are included in the analyses. We did not ask about education level. However, according to NHANES 1999–2000, 74% of Mexican Americans born outside the United States had less than a high school education.

The median energy intake by the FFQ was 115 kcal (5%) higher than that of the 24-hour recalls, whereas the mean energy intake by the FFQ was 265 kcal (12%) higher, indicating the presence of a few outliers (Table 2). Mean nutrient estimates were significantly higher by the FFQ than by the recalls for energy, protein, carbohydrate, total fat, sodium, and calcium, whereas the vitamin C estimate by the FFQ was significantly lower. Nutrient estimates by the two methods were not significantly different for saturated fat, percentage of energy from fat, cholesterol, dietary fiber, iron, or vitamin A.

Correlations between the FFQ and 24-hour recalls (Table 3) were satisfactory (r ≥0.40) for most nutrients (Table 3). Unadjusted correlations exceeded 0.50 for energy, fat, saturated fat, cholesterol, dietary fiber, sodium, total iron, total vitamin A, and total vitamin C. Only vitamin A from food had an unadjusted correlation of less than 0.40. Expressing nutrient estimates as a proportion of energy (nutrient density) lowered most correlations. Correlations using the residual method were similar to the nutrient density method (data not shown). Adjustment for measurement error in the dietary recalls (deattenuation) increased all correlations, ranging from a low of r = 0.36 for vitamin A from food to a high of r = 0.79 for cholesterol.

We examined the ability of the FFQ to identify respondents whose dietary intake did not meet the recommended dietary allowance (RDA) for vitamin C, calcium, and iron or did not meet dietary recommendations for cholesterol and percentage of energy from fat (Table 4). Vitamin A is not reported because the RDA is not expressed in the units available from the recalls and FFQ. Sensitivity ranged from 50% to 77% (median 62%), and specificity from 66% to 88% (median 76%). For example, 69% of people with a cholesterol intake of greater than 300 mg by the recalls (i.e., who failed to meet dietary recommendations) were correctly identified by the FFQ.

## Discussion

With the growing Hispanic population in the United States, and because Hispanics have disproportionately more diet-related diseases such as obesity and diabetes, valid methods for studying their dietary intake are needed. In this study, we showed that an interviewer-administered FFQ yielded unadjusted correlations ranging from r = 0.41 to r = 0.58 and deattenuated correlations ranging from r = 0.43 to r = 0.79 for all nutrients but vitamin A from food.

Relatively few studies have been conducted to evaluate the validity of FFQs among Hispanics. In most of them the FFQ has been self-administered, and results have been modest. Block and DiSogra (3) conducted a validation study of two questionnaires — a modification of the Harvard Prenatal FFQ (4) and a modification of the Block 1992 FFQ. The study was conducted among white, African American, and Hispanic women at clinics of the Special Supplemental Nutrition Program for Women, Infants, and Children (WIC) in California, New York, Ohio, and Texas. Seventy-five Hispanic women were included. The questionnaires were self-administered, covered the previous month, and assessed portion size as "small, medium, or large, compared to other women your age." The reference data were derived from three telephone-administered 24-hour dietary recalls over 1 month. Because the locations included Hispanics nationwide, the Block WIC Spanish version sometimes included two or occasionally three versions of food words that differed among Puerto Rican, Cuban, and Mexican subgroups (e.g., for peas, *ch𝚤́aros* and *guisantes*), which may have been confusing for the Hispanic respondents. For both the Harvard and Block questionnaires, correlations between the FFQs and recalls were poor among Hispanic subjects. The median deattenuated correlation among the six nutrients reported was r = 0.14 for the Harvard questionnaire and r = 0.15 for the Block questionnaire.

Ritenbaugh et al (5) modified a questionnaire to be appropriate for a Hispanic population and conducted a validation study. The resulting Arizona FFQ contained 113 food items, covered the previous 12 months, and assessed portion size using the *small, medium, or large *approach. The questionnaire was self-administered. The reference data were derived from 4 days of food records collected over 4 weeks and obtained after completion of the FFQ. The average unadjusted correlation for energy, fat, and percentage of energy from fat, fiber, and calcium was r = 0.39 for men and r = 0.29 for women. The comparable average unadjusted correlation for these nutrients among men and women combined in our current SWAN questionnaire validation study is r = 0.50 (Table 3).

Kristal et al (6) investigated the validity of the questionnaire used in the Women's Health Trial Feasibility Study in Minority Populations. Hispanic subjects in this study were from Miami, Fla. The data reported here are from the questionnaire obtained 2 weeks after completion of a 4-day food record collected over 1 week. The time frame covered the previous 3 months, and portion size was assessed as small, medium, or large. The questionnaire was self-administered. The unadjusted (r = 0.48) and energy-adjusted (r = 0.53) correlations for calcium are higher than we report, whereas the unadjusted correlations for energy (0.39), fat (0.42), percentage of energy from fat (0.37), saturated fat (0.42), and vitamin C (0.35) are lower than we report for unadjusted correlations (Table 3). Energy-adjusted correlations for fat and saturated fat are also lower than those in Table 3.

Mayer-Davis et al (7) studied the validity of the 114-item Insulin Resistance Atherosclerosis Study FFQ in relation to the mean of eight 24-hour dietary recalls obtained by telephone over 1 year. This is the only other study in which questionnaires were administered by interview, as ours were. The participants were 61 rural Hispanic women in Colorado. The questionnaire was interviewer-administered 1 month after the eight 24-hour recalls had been completed, and it covered the previous 12 months. Portion size was ascertained as "small, medium, or large, compared to other women about your age." Unadjusted correlations for energy (r = 0.27) and macronutrients (fat, r = 0.40; saturated fat, r = 0.38; carbohydrate, r = 0.25) were substantially lower than we report here (Table 3). Correlations for total vitamin A were also lower, whereas correlations for vitamin A from food and vitamin C from food were similar to our correlations. The correlation for total vitamin C (food plus supplements) was higher (r = 0.72) than we report. Energy-adjusted values were not reported separately for the Hispanic participants.

Taren et al (8) studied the validity of the 159-item Southwest FFQ, a modification of the Arizona FFQ (5), among 79 Mexican American and 80 non-Hispanic men and women. The questionnaire was self-administered and covered the previous 12 months. Portion size was ascertained as small, medium, or large. The reference data were derived from 4 days of 24-hour dietary recalls collected by telephone interview over a period of 4 months. The FFQ was administered 2 and 4 weeks after the last 24-hour recall was completed. Unadjusted correlations for macronutrients were lower than those we report, except for calcium, which was similar. Taren et al (8) calculated disattenuated correlations, taking into account not only day-to-day variability in the reference data but also the repeatability coefficient of the FFQ. The disattenuated correlations were higher than our deattenuated correlation for protein (r = 0.68 vs r = 0.61) and vitamin A from food (0.45 vs. 0.36). Other disattenuated correlations were lower than those in Table 3 (mean of energy, macronutrients including protein, and cholesterol, r = 0.55 vs r = 0.78). However, as noted, these disattenuated correlations are not directly comparable to our deattenuated results because they were derived differently.

Stram et al (9) conducted a large validation and calibration study of the Hawaii–Los Angeles Multiethnic Cohort FFQ, which included 259 Hispanics. The time frame covered the previous year, and portion size assessment was facilitated by using photographs of foods. The questionnaire was self-administered. The reference data were derived from three telephone-administered 24-hour dietary recalls collected over 2 months. FFQs were completed 4 to 6 weeks after the last 24-hour recall was finished. Average deattenuated correlations for the amount of energy, protein, fat, saturated fat, and carbohydrate was r = 0.34 for Hispanic men and r = 0.43 for Hispanic women; for the same nutrients, the average deattenuated correlation shown in Table 3 is r = 0.67.

Cullen and Zakeri (10) studied the validity of the 152-food-item Youth/Adolescent Questionnaire among 41 Hispanic seventh- and eighth-grade students. Questions were read to the students in class. The time frame covered the previous year. The reference data were derived from seven self-administered 24-hour dietary recalls, all of which were collected over a period of 21 days following administration of the questionnaire. The deattenuated correlation for energy was r = 0.35 and for percentage of energy from fat was r = 0.04.

Few other investigators have estimated the sensitivity and specificity of estimates from an FFQ for correctly classifying nutrient intake. Only Taren et al (8) have reported such results for Hispanics. In general, their sensitivity results were higher and specificity results were lower than ours. However, these data are not directly comparable, because the RDA cutoffs used by Taren et al were lower for most nutrients than the RDA values we used; for example, for vitamin C, the RDA was 60 mg/day at the time Taren et al conducted the analysis, whereas the RDA values we used were 75 mg/day for women and 90 mg/day for men.

In summary, the validity correlations for the SWAN Spanish FFQ were generally higher than in other studies reviewed here. In all but one of the studies (6), the participants were primarily Mexican American. Methodologic factors may have contributed to the differences in results. For example, in most studies the assessment of portion size differed from ours. One study did not use individually varying portion sizes (10); five studies used the *small, medium, or large* approach (4-8). Only one study (9) used portion-size graphics as we did. Our use of the visual portion-size graphic could have led to more accurate estimations of quantities consumed.

The nature of the reference data is another methodologic factor that can influence the apparent validity of an FFQ. A large number of days of recalls or records will usually result in a higher estimate of an FFQ's validity simply because of improvements in the quality of the reference data (23). However, most studies, including ours, used 3 or 4 days of diet recalls or records as the reference data, so this does not explain the differences in results. Only Mayer-Davis et al (7) obtained a higher number, eight 24-hour recalls over 1 year, but this did not generally produce higher correlations than ours.

The nature of the reference data may also have affected the correlations for vitamin A in this and previous studies. In our study, only food vitamin A had a correlation of r < 0.40. The day-to-day variability of dietary vitamin A is well-established (18). Beaton et al (24) and others have noted that a very large number of days of dietary data may be required to obtain reasonably precise intake estimates of vitamin A. Thus, the use of 3 days as the reference data may have led to lower correlations for vitamin A.

A notable methodologic factor is the way in which the questionnaire was administered. Although we administered the questionnaire by interview, all but one (7) of the studies discussed obtained questionnaire responses by self-administration. Kristal et al (6) and others (4,7) have found that validity correlations based on self-administered questionnaires tend to be lower among people with lower levels of education. The average education level among Hispanic adults in the United States is substantially lower than that of African American and non-Hispanic white adults. In the NHANES 1999–2000 data, 19.9% of white adults had less than a high school education, and 43.6% of African American adults had less than a high school education. In contrast, 50% of Hispanic adults had less than a high school education. Among Mexican Americans, 58% had less than a high school education; among Mexican Americans born outside the United States, 74% had less than a high school education. Thus, we suggest that part of the reason we found generally higher correlations is that our FFQ was administered by interview, whereas most other studies have used self-administered FFQs. This suggests that in this population, better information about dietary behavior may be obtained by interview than by self-administration. However, our correlations are generally higher than those reported by Mayer-Davis et al (7), who also administered the FFQ by interview.

The SWAN questionnaire tended to overestimate calcium intake and had a lower sensitivity for detecting calcium intakes below the adequate intake (AI) (22). The likely reason for this is that the SWAN questionnaire contained three milk items (whole, 2%, and low fat), each as a separate item. It is likely that some respondents reported their milk intake in more than one milk item. This potential respondent error is prevented in subsequent versions of the Block FFQ (16).

The food list used in the SWAN questionnaire was based on a 103-item core list developed from representative national data and administered to all ethnic groups in the SWAN study. For Hispanic respondents, it was augmented by only nine items deemed especially appropriate for Hispanics. It is interesting that this food list apparently was quite appropriate even for our sample of Mexico-born Spanish-speaking participants. We have shown in other work that ethnic-specific foods contribute only modestly to the estimates of nutrient intake for ethnic groups in the United States (25).

A limitation of our validation study is the fact that the participants were almost all Mexican Americans. It would have been desirable to have had other Hispanic subgroups represented in this study. However, four of the nine Hispanic items added to the core food list are important nutrient contributors in the diets of Cuban and Puerto Rican Americans but not for Mexican Americans. Thus, we believe the questionnaire is valid for a range of Hispanic subgroups.

The unadjusted correlations in Table 3 represent the simple relationships between nutrient estimates from the FFQ and the reference method. In some studies, the FFQ estimate of the absolute level of the nutrient is of interest. The sensitivity analysis is an example of such a situation. Often researchers wish to know what the respondent is actually consuming, not simply whether the nutrient intake is proportional to energy intake or how it ranks in comparison with the intake of other participants. For example, in some situations, we would not deem a man's vitamin C intake to be adequate at 45 mg/day (RDA = 90 mg/day) simply because his energy intake was low. We would actually want to know whether his vitamin C intake was 90 mg. In this circumstance, the nutrient estimates unadjusted for energy would be the ones of interest.

The unadjusted nutrient estimate may also be used appropriately in energy-adjusted analyses in which the nutrient is being treated as a continuous variable. For example, in a regression model such as the following:

Body Mass Index = Calories + Saturated Fat,

in which saturated fat is a continuous variable, the unadjusted nutrient estimate may be used because the coefficient for saturated fat is identical regardless of whether the unadjusted variable or the residual is used. Thus, in such situations the unadjusted nutrient estimate is appropriate, and the unadjusted correlations are of interest. If on the other hand the nutrient variable is categorized (e.g., divided into quartiles), it may be argued that the residualized variable should be used (19).

Deattenuated correlations attempt to approximate what the correlation of the FFQ with "truth" would be if within-person variability in the recalls could be rendered unimportant by having an infinite number of days in the reference data. However, all we really know is that the observed correlation probably underestimates the unobservable "true" correlation; it may be imprudent to suppose that we know how much it underestimates the correlation. Deattenuation can make an obviously poor correlation into an improbably good correlation, simply because of high within-person variability. Therefore, deattenuated correlations may give an erroneous sense of confidence in the FFQ and should be considered with caution.

Full-length dietary assessment tools are needed to investigate optimally the role of dietary patterns in health and disease. However, in some situations the logistics or respondent burden make it impractical. In a separate component of the study reported here, we also developed and tested the reliability of brief screening instruments for evaluating intake of fruits and vegetables and fat in Hispanics (26). These tools may be useful if a full questionnaire is not needed or practical, and additional research into their validity is warranted.

Our study demonstrates that the SWAN FFQ may be a useful tool for dietary assessment among Mexican Americans when administered by interview. We believe it would have similar validity among other Hispanic subgroups. It will be important in the future to investigate whether user-friendly computer-assisted interview methods can improve the success of self-administration by Hispanics.

## Figures and Tables

**Table 1:** Characteristics of Participants (n = 89) in a Validation Study of a Spanish-Language Food Frequency Questionnaire (FFQ),^a^  Berkeley, Calif, 2000

**Characteristic**	**Value**
**Sex, %**	
Male (n = 37)	41.6
Female (n = 52)	58.4
**Birthplace, %**	
Mexico (n = 82)	92.1
South America (n = 2)	2.3
United States (n = 5)	5.6
**Age, y**	
Mean (SD)	36.8 (14.7)
Range	18-71

a The FFQ used was the Study of Women's Health Across the Nation questionnaire.

**Table 2:** Comparison of Nutrient Intake by Food Frequency Questionnaire (FFQ) and 24-Hour Recalls (n = 89), Berkeley, Calif, 2000

**Nutrient^a^**	**Recalls**		**FFQ**		***P* Value^b^**

	**Mean (SD)**	**Median**	**Mean (SD)**	**Median**	
Energy, kcal	2209.3 (615.4)	2259.5	2474.0 (1016.3)	2374.9	.007
Protein, g	83.6 (27.2)	80.4	94.1 (36.2)	87.8	.005
Carbohydrate, g	300.1 (90.1)	296.0	336.0 (153.7)	312.2	.02
Fat, g	77.5 (30.6)	74.5	85.3 (39.7)	80.3	.04
Saturated fat, g	26.7 (12.1)	25.5	27.6 (14.9)	26.1	.52
Cholesterol, mg	308.1 (163.7)	271.0	324.8 (173.0)	292.9	.33
Dietary fiber, g	22.5 (10.0)	20.5	22.8 (10.8)	21.4	.72
Sodium, mg	2788.8 (962.5)	2750.4	3065.9 (1289.5)	2967.5	.04
Calcium, mg	892.0 (370.0)	817.9	1255.2 (689.6)	1147.1	<.001
Iron, mg	15.9 (5.4)	15.3	16.0 (6.7)	14.9	.87
Vitamin A, RE	1122.7 (783.1)	927.3	1137.9 (851.6)	785.8	.89
Vitamin C, mg	225.6 (302.4)	158.5	144.2 (96.9)	118.1	<.001

RE indicates retinol equivalents.

a Nutrients for which at least 90% of the foods on the Food Processor version 7.0 database (ESHA Research Inc, Salem, Ore) had a value for the nutrient.

b Significance of the difference between mean recall and FFQ estimates.

**Table 3:** Correlations Between Food Frequency Questionnaire (FFQ) Estimates and the Mean of Three 24-Hour Dietary Recalls^a^ (n = 89), Berkeley, Calif, 2000

**Nutrient^b^**	**Pearson Correlations**		

	**Unadjusted**	**Energy Adjusted**	**Deattenuated^c^**
Energy, kcal	0.52	—	0.69
Protein, g	0.49	0.30	0.61
Carbohydrate, g	0.47	0.46	0.61
Fat, g	0.58	0.42	0.78
Percentage of energy from fat	0.41	—	0.56
Saturated fat, g	0.54	0.45	0.68
Cholesterol, mg	0.58	0.47	0.79
Dietary fiber, g	0.54	0.67	0.68
Sodium, mg	0.53	0.10	0.75
Calcium from food only, mg	0.43	0.42	0.58
Total calcium, including supplements, mg	0.43	—	0.43
Iron from food only, mg	0.48	0.31	0.67
Total iron, including supplements, mg	0.53	—	0.54
Vitamin A from food only, RE	0.27	0.33	0.36
Total vitamin A, including supplements, RE	0.52	—	0.53
Vitamin C from food only, mg	0.41	0.40	0.52
Total vitamin C, including supplements, mg	0.56	—	0.55

RE indicates retinol equivalents.

a The relationship between the FFQ and the 24-hour dietary recall nutrient values was estimated using Pearson correlation coefficients.

b Only nutrients for which at least 90% of the foods on the Food Processor database had a value for the nutrient. Log-transformed where needed to improve normality.

c Adjusted for measurement error in dietary recalls.

**Table 4:** Ability of Food Frequency Questionnaire (FFQ) to Identify People Not Meeting RDA for Nutrients or Recommendations for Cholesterol and Fat (n = 89), Berkeley, Calif, 2000

**Criterion**	**Percent of Sample Below the Criterion**		**Sensitivity, %^c^**	**Specificity, %^d^**

	**In Recall,^a^ %**	**In** **FFQ,^b^ %**		
Below RDA				
Vitamin C	18	19	50	88
Iron	63	61	77	67
Calcium	70	42	50	78
Cholesterol <300 mg/day	44	44	69	76
Percentage of energy from fat <30%	61	55	62	66

RDA indicates recommended dietary allowance.

a Percentage of respondents who failed to meet the criterion, by three 24-hour recalls.

b Percentage of respondents who failed to meet the criterion, by FFQ.

c Of those identified by recalls as failing to meet the recommendations, the percentage correctly identified by FFQ.

d Of those identified by recalls as meeting recommendations, the percentage correctly identified by FFQ.
